# Development of an explicit tool assessing potentially inappropriate medication use in Hong Kong elder patients

**DOI:** 10.1186/s12877-021-02024-0

**Published:** 2021-02-02

**Authors:** Huanyu Zhang, Eliza LY Wong, Eng-kiong Yeoh, Bosco HM Ma

**Affiliations:** 1 Centre for Health Systems and Policy Research, The Jockey Club School of Public Health and Primary Care, Faculty of Medicine, The Chinese University of Hong Kong, Prince of Wales Hospital, Shatin, New Territories Hong Kong; 2 Department of Medicine and Therapeutics, Prince of Wales Hospital, Shatin, New Territories, Hong Kong

**Keywords:** Potentially inappropriate medication, Explicit criteria, Delphi method, Older adults, Hong Kong

## Abstract

**Background:**

Potentially inappropriate medication (PIM) use has adverse effects on health, particularly in elder patients. Various country-specific explicit criteria have been developed to measure the appropriateness of prescribing worldwide. However, it is difficult to apply the criteria developed from other regions to measure and guide the local prescribing practice in Hong Kong. This study aims to develop a Hong Kong-specific PIM assessing tool from previously published criteria and validate this tool using the modified Delphi method.

**Methods:**

A disease-oriented Hong Kong-specific preliminary PIM list was developed based on nine sets of reference criteria selected from a literature review. Any medication or medication class appeared in at least two sets of the reference criteria as well as its related medical conditions were selected as PIM candidates. After examining the availability of PIM candidates by the Hong Kong Hospital Authority drug formulary, the Hong Kong-specific preliminary PIM list was validated by a two-round of modified Delphi process. Eight experts from different specialties were invited to rate the degree of inappropriateness of each PIM candidate using a five-point Likert scale. The experts were also encouraged to propose therapeutic alternatives and new PIM candidates not covered by the preliminary PIM list. The PIM candidates that the expert panel didn’t reach consensus on were excluded from the final Hong Kong-specific PIM list.

**Results:**

After two rounds of the Delphi process, eight PIM candidates remained questionable and thus were excluded from the PIM list. The final Hong Kong-specific PIM list included a total of 164 statements applicable to older adults aged 65 years or above, among which 77 were under PIMs independent of diagnoses, and 87 were under PIMs considering specific medical conditions.

**Conclusions:**

The Hong Kong-specific PIM list can be used as a quality measure and an educational tool to improve the local prescribing quality. Further studies should validate its association with adverse health outcomes in clinical and research settings.

**Supplementary Information:**

The online version contains supplementary material available at 10.1186/s12877-021-02024-0.

## Background

Potentially inappropriate medication (PIM) use, generally occurring when patients are prescribed drugs with an unfavorable balance of benefits and risks, is a preventable problem [1]. PIM use can lead to adverse drug events, which elevate the risk of poor quality of life and high health care costs [2]. Older adults are particularly at high risk for PIM use due to physiologic changes in pharmacokinetics and pharmacodynamics [3]. Moreover, a great number of older adults suffer from comorbid diseases and are prescribed multiple medications [4]. Several studies revealed that polypharmacy, which referred to the use of five and more prescribed drugs at the same time, was associated with greater risk of PIM use [3, 5, 6]. In Hong Kong, people aged 65 years or older accounted for 17.9% of the total population in 2018 and this proportion was projected to be 33.7% by 2066 [7]. To present a wider perspective, in 2019, the proportions of older adults in the populations of North America and Europe were estimated to be 16.4 and 18.8%, respectively [8]. Therefore, it is important to identify PIMs among the older adults in Hong Kong to minimize drug-related issues and improve health outcomes.

With an aging population, appropriateness of prescribing has become a public health concern worldwide and thus, various tools have been developed to assess the quality of prescribing. These measures can be generally categorized into explicit (criterion-based) and implicit (judgment-based) tools [9]. Explicit tools are typically firm standards developed from published literature, expert opinions and consensus techniques [10]. They are usually drug or disease oriented and can be applied to large samples of people to measure the appropriateness of prescribing from a population level [11]. By contrast, implicit tools can evaluate the appropriateness of prescribing from an individual level depending on a health professional’s judgement. However, it is time-consuming to apply implicit tools in clinical practice and they have low reliability and reproducibility [8, 9]. As a systematic approach, explicit tools are therefore more favored in real world practice.

Older adults are usually excluded from clinical trials, thus lacking evidence on balancing risks and benefits of therapeutic agents in this population [12]. Under this circumstance, consensus techniques such as Delphi methods, are acceptable for use in developing explicit assessing tools despite its limitations of subjective evaluations [13]. The first explicit tool for assessing PIM use in older adults was the Beers criteria created based on Delphi methods in the US in 1991 [14] and updated on a three-year cycle since 2012 [15]. Although the Beers criteria were widely used to assess the quality of prescribing globally, several researchers indicated the difficulty of adapting the Beers criteria into the local situation because of contextual differences in terms of licensed drugs, clinical practice and health system regulations [16–18]. Accordingly, it is unlikely to transfer explicit indicators from one country to another without going through a process of adaption and modification [7]. In recent years, tools for assessing prescribing appropriateness have intensified in different countries across Europe, North America and Asia [19]. However, limited overlap could be observed between different PIM lists and some of them were lack of special considerations of use and therapeutic alternatives to PIMs [19].

In Hong Kong, we recently conducted a study to assess the prevalence of PIM use in elder patients aged 65 years and older visiting general outpatient clinics, using the main subsets of the 2015 Beers criteria. The prevalence estimates ranged from 55.6% in 2006 to 47.5% in 2014, which were relatively higher than those reported in western countries [6, 20, 21], ranging from 2.9 to 43.3% assessed by the Beers criteria. We also found that only 60% of the statements from the Beers criteria that we addressed in the study were available in the drug formulary from Hospital Authority (HA), which is a statutory body to manage all public hospitals and clinics in Hong Kong. To enhance the comprehensiveness of quality criteria to evaluate medication use in the context of Hong Kong, it is necessary to develop a Hong Kong-specific explicit assessing tool. To our knowledge, up to now, no Hong Kong-specific PIM lists have been established or validated yet. The aim of this study was to develop a Hong Kong-specific PIM assessing tool on the basis of published criteria in the literature and input from the local context using the modified Delphi method.

## Methods

### Development of a preliminary PIM list

The first step in developing the Hong Kong-specific PIM criteria was to establish a preliminary drug list based on existing criteria in the literature. A literature search was conducted to identify explicit tools assessing PIM use on the PubMed database from January 1991 to April 2019 internationally and consequently, 40 sets of validated explicit criteria were identified. Explicit criteria that 1) applied to the general population aged 65 years and older, 2) used the literature review on safety and efficacy of drug use as an evidence base, and 3) described the methods of development and validation for the criteria, were taken into account. Regarding the criteria that were updated occasionally, only the latest versions of PIM lists were considered and the early versions of the criteria were eliminated from the study. Consequently, the following nine sets of explicit criteria were included as reference criteria in the current study: the McLeod criteria [22], the Rancourt criteria [23], the Lindblad criteria [24], the Laroche criteria [17], the Winit-Watjana criteria [25], the Norwegian General Practice [26], the PRISCUS criteria [12], the version 2 of the Screening Tool of Older Person’s Prescription (STOPP) [27], and the 2015 version of the Beers criteria [28].

The structures of the nine sets of reference criteria were heterogeneous, with the classification system of criteria statements as disease-oriented or/and drug-oriented. The evaluated aspects of the reference criteria are summarized in Table 1. The most common evaluated aspects in the nine sets of reference criteria included generally avoided drugs independent of diagnoses (7/9), dosage (6/9), disease-drug interactions (6/9), and drug-drug interactions (7/9). After reviewing the structures of the reference criteria, the evaluation of dosage was found to be rarely classified as a separate category of PIM use in the reference criteria. Furthermore, the lists of drug-drug interactions in the reference criteria were mostly selective and not comprehensive. Therefore, the current study was intended to exclude drug-drug interactions and develop a disease-oriented preliminary PIM list. The structure of the Hong Kong-specific preliminary PIM list was formulated using two common categories of PIMs from the reference criteria, namely, generally avoided drugs independent of diagnoses, and disease-drug interactions.

**Table 1:** Summary of evaluated aspects in the nine sets of reference criteria

List name	Country	Independent of diagnosis (7/9)	Dosage (6/9)	Duration of therapy (4/9)	Disease-drug interactions (6/9)	Drug-drug interactions (7/9)	Duplication (3/9)	Alternative therapies (4/9)	Special consideration of use (4/9)
McLeod criteria	Canada			✓	✓	✓		✓	✓
Rancourt criteria	Canada	✓	✓	✓		✓	✓		
Lindblad criteria	USA				✓				
Laroche criteria	France	✓	✓		✓	✓	✓	✓	
Winit-Watjana criteria	Thailand	✓			✓	✓			
NORGEP	Norway	✓	✓			✓			
PRISCUS	Germany	✓	✓					✓	✓
STOPP version 2	Ireland	✓	✓	✓	✓	✓	✓	✓	✓
Beers criteria	USA	✓	✓	✓	✓	✓			✓

Based on the structure of the preliminary PIM list, the operational definitions of PIM use in the current study are described as follows: 1) medications or medication classes that should be generally avoided independent of diagnosis or conditions and 2) medications or medication classes that should not be used in older adults known to have specific medical conditions. Other evaluated aspects of PIM use including dosage, duration of therapy, alternative therapies and special considerations of use were supplemented by experts’ comments during the Delphi process. Following the operational definition of PIM use in this study, seven sets of reference criteria included generally avoided drugs independent of diagnoses and six sets of criteria included disease-drug interactions. After extracting all related statements from the reference criteria, 279 medications/classes under generally avoided drugs independent of diagnoses and 115 medications/classes considering specific medical conditions were identified.

To prepare a manageable amount of PIM statements for further evaluations in the Delphi process, any medication or medication class appeared in at least two sets of the reference criteria (if appeared in two sets of criteria, one of which must be the Beers criteria) and its related medical conditions were selected from relevant reference criteria as candidate PIMs for each category of PIM use. We used the Beers criteria to select medications that appeared in two sets of criteria because the Beers criteria were considered as the golden standard for PIM assessment and many country-specific explicit criteria were developed based on the Beers criteria [19]. The availability of candidate PIMs was examined by the Hospital Authority (HA) drug formulary issued in April 2018. The candidates not covered by the HA drug formulary were excluded from the preliminary PIM list. Meanwhile, all the individual medications belonging to each medication class were also identified from the HA drug formulary. The medications/classes were identified by the Anatomical Therapeutic Chemical (ATC) classification system codes to enhance the standardization of PIM candidates. After applying the inclusion and exclusion criteria, the final Hong Kong-specific preliminary PIM list included 168 candidates, 78 of which were developed under PIMs independent of diagnoses, and 90 were developed under PIMs considering specific medical conditions for further questioning in the Delphi process.

### Delphi method

The preliminary PIM list was validated by a two-round modified Delphi process. Experts from different specialties were recruited by invitation emails and personal communication, all involved with practice or research in medication issues for elder patients for at least five years. A face-to-face meeting was also convened with six potential experts one by one to brief them on the study background and objectives. After the briefing meeting, five experts decided to participate in the project. Invitation emails were also disseminated to the Hong Kong Geriatric Society (HKGS). Four geriatric physicians from the HKGS replied for a further in-person meeting and three of them finally decided to take part in the study after the meeting. Overall, a total of eight (80%) experts agreed in participating in the project seeking to develop the Hong Kong-specific PIM list. On the basis of the preliminary PIM list, a five-point Likert scale was designed to construct the questionnaire for collecting experts’ ratings and comments during the Delphi process. The questionnaire was examined for wording and content validity by three qualified experts.

The first round of the Delphi process began in August, 2019. The experts were asked to rate the degree of inappropriateness of each candidate statement from a score of 1 (strongly disagree) to 5 (strongly agree). A score of 3 was neutral or undecided. Furthermore, the experts were encouraged to provide comments on any exception or restriction in terms of indication, dose, route of administration, or therapy duration to make each candidate statement more accurate. They were also asked to propose therapeutic alternatives to the PIMs independent of diagnoses and suggest new PIM candidates not included in the questionnaire.

After the first round of questioning, the experts’ answers were presented as median with interquartile ranges (IQR) for each candidate PIM. The statements with medians ≥3.5 and IQR ≤ 1.5 were included in PIM use, whereas the statements with medians < 3.0 were excluded from PIM use [25]. The statements with 3.0 ≤ medians < 3.5 or IQR > 1.5 were regarded as questionable PIMs that required further evaluations in the second round along with the new candidate PIMs suggested by the expert panel [25]. The second round of the Delphi process was conducted in October, 2019. All the experts received the second-round questionnaire together with the panel’s ratings and comments from the first round. The experts’ feedbacks in the second round were evaluated using the same procedure.

## Results

Eight experts participated in the first round of the Delphi process. One expert with a pharmacy background quit during the second round of questioning due to illness. The characteristics of eight experts are shown in Table 2. All the experts worked in public hospitals or universities, and the majority of them were males. The expert panel had specialties in geriatric medicine, pharmacy, family medicine, and internal medicine, and half of them were geriatric physicians. Most of the experts had a working experience related to medication issues for elder patients longer than 10 years (50% longer than 20 years).

**Table 2:** Characteristics of the expert panel

Characteristics	No. of experts (%)
**Male**	6 (75.0)
**Working field(s)**	
Geriatric medicine	6 (75.0)
Pharmacy	3 (37.5)
General practice/Family medicine	1 (12.5)
Internal medicine	1 (12.5)
**Profession**	
Pharmacists	3 (37.5)
Geriatric physician	4 (50.0)
General practitioner	1 (12.5)
**Years of experience related to medication issues for elder patients**	
5 ~ 10 years	1 (12.5)
11 ~ 15 years	3 (37.5)
> 20 years	4 (50.0)
**Working place**	
University	2 (25.0)
Public hospital	6 (75.0)

In the first round of the Delphi process, 117 (69.6%) candidate statements were designated as PIM use whereas 8 (4.8%) statements were designated as non-PIM use and dropped from the study. Forty-three (25.6%) candidates were classified as questionable PIM use, which required further evaluations in the second round. Furthermore, three new indications of the PIMs listed in the preliminary list were suggested by the expert panel to be included in the second round of rating. According to the ATC classification system, terazosin is classified as a urological drug while prazosin and doxazosin are classified as antihypertensives; however, they all belong to alpha-adrenoreceptor antagonists and can be indicated as either a urological or an antihypertensive drug based on the expert panel’s comments. The indications not included in the first round of questioning were rated again in the second round. The expert panel also proposed nine new PIM candidates not incorporated in the preliminary list, seven of which were under PIMs independent of diagnoses, and two were under PIMs considering specific medical conditions. Furthermore, the experts’ comments in terms of rationales, restrictions, and exceptions for each PIM candidate were consolidated and expanded based on the reference criteria. Consequently, a total of 55 candidates, including 44 in the current list plus 12 new suggestions, was brought forward to the next round of the Delphi voting.

**Fig. 1:**
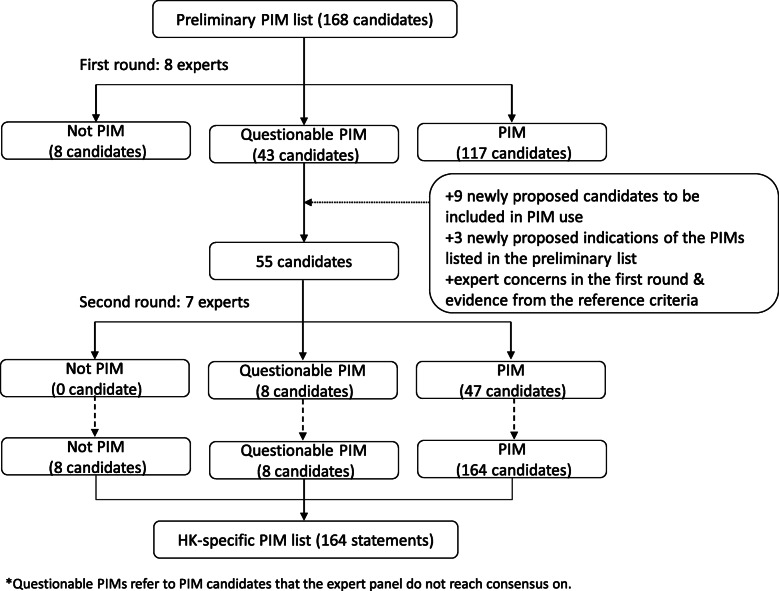
Flowchart of the Delphi process

In the second round of questioning, 47 (85.5%) candidates were rated as PIM use while 8 (14.5%) candidates remained questionable PIM use (Table 3). Among the eight questionable candidates, one candidate was rated with the IQR value greater than 1.5 whereas the other seven candidates were rated with the median value lower than 3.5. The eight questionable PIM candidates were excluded from the Hong Kong-specific PIM criteria due to the large discrepancy or low median score based on the experts’ ratings. The flowchart of the Delphi process is shown in Fig. 1. After two rounds of the Delphi process, the final Hong Kong-specific PIM list included 164 statements, among which 77 were under PIMs independent of diagnoses (Table 4), and 87 were under PIMs considering specific medical conditions (Table 5). The full list of medications or medication classes with ATC codes included in PIMs considering specific medical conditions are presented in Additional file 1. The main concerns and the possible therapeutic alternatives for the Hong Kong-specific PIM list are presented in Additional file 2.

**Table 3:** Questionable PIMs that the expert panel did not reach consensus

PIMs independent of diagnoses			
**Medication class**	**Medication**	**Median**	**IQR**
**Antithrombotic agents**	Dipyridamole	3	2.50 ~ 3.50
**Antidepressants**	Dosulepin (dothiepin)	4	2.50 ~ 4.50
	Fluoxetine	3	2.00 ~ 3.50
**Analgesics**	Acetaminophen	3	2.50 ~ 4.00
	Methyl salicylate topical ointment	3	3.00 ~ 4.00
**PIMs considering specific medical conditions**			
**Disease/Syndrome**	**Medication class/Medication**	**Median**	**IQR**
**Blood clotting disorders**	Clopidogrel	3	3.00 ~ 4.00
	Dipyridamole	3	3.00 ~ 4.00
**Falls**	SSRIs	3	3.00 ~ 4.00

**Table 4:** Hong Kong-specific potentially inappropriate medication list: independent of diagnosis (results of the Delphi process)

Medication class	Medications	ATC code	Median	IQR
**Antispasmodics**	Propantheline	A03AB05	5	3.50 ~ 5.00
	Atropine	A03BA01	5	3.50 ~ 5.00
	Metoclopramide	A03FA01	3.5	2.75 ~ 4.00
**Long-acting sulfonylureas**	Glipizide	A10BB07	4	4.00 ~ 4.50
**Cardiac glycosides**	Digoxin	C01AA05	4	3.00 ~ 4.00
**Antiarrhythmics**	Disopyramide	C01BA03	4	3.00 ~ 4.00
	Amiodarone	C01BD01	4	3.00 ~ 4.00
**Antihypertensives**	Methyldopa	C02AB	4	3.75 ~ 4.25
	Clonidine	C02AC01	4	3.75 ~ 4.25
	Prazosin	C02CA01	4	2.75 ~ 4.00
	Doxazosin	C02CA04	4	4.00 ~ 5.00
	Terazosin	G04CA03	4	4.00 ~ 5.00
**Calcium channel blockers**	Nifedipine	C08CA05	4	4.00 ~ 5.00
**Androgens**	Testosterone	G03BA03	4	4.00 ~ 4.00
**Estrogens**	Estradiol	G03CA03	3.5	3.00 ~ 4.00
	Tibolone	G03CX01	3.5	3.00 ~ 4.00
**Urologicals**	Oxybutynin	G04BD04	3.5	2.75 ~ 4.00
	Terazosin	G04CA03	4	4.00 ~ 4.00
	Doxazosin	C02CA04	4	4.00 ~ 4.00
	Prazosin	C02CA01	4	4.00 ~ 4.00
**Antibacterials for systemic use**	Nitrofurantoin	J01XE01	4	4.00 ~ 5.00
**Antiinflammatory and antirheumatic products**	Indometacin	M01AB01	4	3.00 ~ 4.00
	Sulindac	M01AB02	4	3.00 ~ 4.00
	Diclofenac	M01AB05	4	4.00 ~ 4.00
**Antiinflammatory and antirheumatic products**	Ketorolac	M01AB15	4	3.75 ~ 4.00
	Piroxicam	M01AC01	4	3.75 ~ 4.00
	Meloxicam	M01AC06	4	3.75 ~ 4.00
	Ibuprofen	M01AE01	4	4.00 ~ 4.00
	Naproxen	M01AE02	4	3.00 ~ 4.00
	Mefenamic acid	M01AG01	4	3.00 ~ 4.00
	Celecoxib	M01AH01	4	4.00 ~ 4.00
	Etoricoxib	M01AH05	4	4.00 ~ 4.00
**Muscle relaxants**	Orphenadrine	M03BC01	4	4.00 ~ 4.00
	Baclofen	M03BX01	4	4.00 ~ 4.00
	Tolperisone	M03BX04	4	3.50 ~ 4.00
**Antipsychotics**	Chlorpromazine	N05AA01	4	4.00 ~ 4.25
	Prochlorperazine	N05AB04	3.5	2.75 ~ 4.00
	Trifluoperazine	N05AB06	4	3.75 ~ 4.25
	Haloperidol	N05AD01	4	4.00 ~ 4.00
	Ziprasidone	N05AE04	4	3.50 ~ 4.00
	Pimozide	N05AG02	4	4.00 ~ 4.50
	Clozapine	N05AH02	3.5	3.00 ~ 4.00
	Olanzapine	N05AH03	4	3.50 ~ 4.00
	Quetiapine	N05AH04	4	3.50 ~ 4.00
	Risperidone	N05AX08	4	3.50 ~ 4.00
	Aripiprazole	N05AX12	4	3.50 ~ 4.00
	Paliperidone	N05AX13	4	3.50 ~ 4.00
**Benzodiazepines**	Clonazepam	N03AE01	4	2.75 ~ 4.25
	Diazepam	N05BA01	4	2.75 ~ 4.25
	Chlordiazepoxide	N05BA02	4	2.75 ~ 4.25
	Lorazepam	N05BA06	4	3.50 ~ 5.00
	Bromazepam	N05BA08	4	2.75 ~ 4.25
	Clobazam	N05BA09	4	2.75 ~ 4.25
	Alprazolam	N05BA12	3.5	2.75 ~ 4.00
	Nitrazepam	N05CD02	4	2.75 ~ 4.25
**Benzodiazepines**	Flunitrazepam	N05CD03	4	2.75 ~ 4.25
	Triazolam	N05CD05	3.5	2.75 ~ 4.00
	Midazolam	N05CD08	3.5	2.75 ~ 4.00
**Anxiolytics**	Hydroxyzine	N05BB01	4	3.00 ~ 4.00
**Hypnotics and sedatives**	Zopiclone	N05CF01	4	3.00 ~ 4.50
	Zolpidem	N05CF02	4	3.00 ~ 4.50
**Antidepressants**	Imipramine	N06AA02	4	4.00 ~ 5.00
	Clomipramine	N06AA04	4	4.00 ~ 5.00
	Trimipramine	N06AA06	4	4.00 ~ 5.00
	Amitriptyline	N06AA09	4	3.50 ~ 5.00
	Nortriptyline	N06AA10	4	3.50 ~ 5.00
	Doxepin	N06AA12	4	3.75 ~ 5.00
**Antihistamines**	Chlorpheniramine	R06AB04	3.5	2.75 ~ 4.25
	Cyproheptadine	R06AX02	3.5	2.75 ~ 4.25
	Dexchlorpheniramine	R06AB02	3.5	2.75 ~ 4.25
	Diphenhydramine	R06AA02	4	2.75 ~ 4.25
	Promethazine	R06AD02	3.5	2.75 ~ 4.25
**New PIMs suggested by the expert panel**				
**Analgesics**	Acetaminophen	N07BC02	3	2.50 ~ 4.00
	Methyl salicylate topical ointment	A06AG01	3	3.00 ~ 4.00
	Methadone	A06AD15	4	4.00 ~ 4.50
**Laxatives**	Sodium phosphate enema	M05BA	4	4.00 ~ 4.00
	Polyethylene glycol electrolyte powder	N02AA59	4	3.50 ~ 4.00

**Table 5:** Hong Kong-specific potentially inappropriate medication list: considering specific medical conditions (results of the Delphi process)

Disease/Syndrome	Medication class/Medication	Median	IQR
**Blood and blood-forming organs**			
Blood clotting disorders	NSAIDs	4	3.00 ~ 4.00
	Asprin	3.5	2.75 ~ 4.00
**Cardiovascular**			
Syncope	AChEIs	4	3.00 ~ 4.00
	Thioridazine	4	4.00 ~ 4.25
	TCAs	4	3.75 ~ 5.00
	Alpha-adrenoreceptor antagonists	4	3.75 ~ 4.25
	Chlorpromazine	4	4.00 ~ 5.00
	Methyldopa	4	4.00 ~ 4.25
Heart failure	Thiazolidinediones	4	4.00 ~ 4.25
	NSAIDs	4	3.75 ~ 4.25
	Nondihydropyridine CCBs	4	3.50 ~ 4.50
Heart block	TCAs	5	4.00 ~ 5.00
	Beta blocking agents	5	4.00 ~ 5.00
Cardiac arrhythmia	Antipsychotics	4	3.00 ~ 4.00
**Central nervous system**			
Delirium	Sedative hypnotics	4	3.00 ~ 4.25
	Benzodiazepines	4	4.00 ~ 5.00
	Anticholinergics	4	4.00 ~ 4.25
	Antipsychotics	3.5	2.75 ~ 4.00
	Corticosteroids	3.5	3.00 ~ 4.00
Parkinson disease	Antipsychotics	3.5	3.00 ~ 4.00
	Metoclopramide	4	4.00 ~ 4.25
Dementia/ Cognitive impairment	Benzodiazepines	4	2.75 ~ 4.25
	Anticholinergics	4	3.50 ~ 4.25
	TCAs	4	3.50 ~ 4.25
	Antipsychotics	3.5	2.75 ~ 4.00
Falls	Sedative hypnotics	4	3.00 ~ 4.25
	Thioridazine	4	3.75 ~ 4.25
	Benzodiazepines	4	4.00 ~ 4.50
	TCAs	4	3.50 ~ 5.00
	Antipsychotics	4	3.50 ~ 4.25
	Opioids	4	4.00 ~ 4.50
Epilepsy/Seizures	Bupropion	4	3.75 ~ 4.00
	Thioridazine	4	4.00 ~ 4.00
	Antipsychotics	3.5	3.00 ~ 4.00
Behavioural and psychological symptoms of dementia	Antipsychotics	3.5	2.50 ~ 4.00
Depression	Methyldopa	4	3.75 ~ 4.25
Lewy body disease	Antipsychotics	4	3.75 ~ 4.00
Sleep apnea syndrome	Benzodiazepines	4	3.75 ~ 4.00
**Circulatory system**			
Postural hypotension	TCAs	4.5	4.00 ~ 5.00
	Dihydropyridine CCBs	4	3.50 ~ 4.00
	Alpha-adrenoreceptor antagonists	4.5	3.75 ~ 5.00
	Chlorpromazine	4.5	4.00 ~ 5.00
Hypertension	NSAIDs	4	4.00 ~ 4.50
Raynaud disease	Beta blocking agents	4	3.75 ~ 4.00
Postural hypotension	Thioridazine	4	4.00 ~ 4.25
Venous thromboembolism	Oestrogens	4	4.00 ~ 4.25
**Endocrine, nutritional and metabolic system**			
Hypokalaemia	Thiazide diuretics	4	3.75 ~ 4.00
Hyponatraemia	SSRIs	3.5	3.00 ~ 4.00
Hyponatraemia	Thiazide diuretics	4	3.75 ~ 4.00
Hyperkalaemia	AChEIs	3.5	3.00 ~ 4.00
Hypercalcaemia	Thiazide diuretics	4	3.00 ~ 4.00
Diabetes	Corticosteroids	4	4.00 ~ 4.00
Diabetic hypoglycemia	Beta blocking agents	3.5	3.00 ~ 4.00
**Eye and adnexa**			
Glaucoma	Anticholinergics	4	4.00 ~ 4.00
	TCAs	4	4.00 ~ 5.00
**Gastrointestinal System**			
Chronic constipation	Anticholinergics	4	4.00 ~ 4.25
	TCAs	4	4.00 ~ 5.00
	Methyldopa	4	3.00 ~ 4.00
	Opioids	4	3.75 ~ 4.25
	CCBs	3.5	3.00 ~ 4.00
**Kidney and urinary tract**			
Chronic kidney disease	NSAIDs	5	4.75 ~ 5.00
Peptic ulcer disease	NSAIDs (Non-COX-2 selectvie agents)	4.5	4.00 ~ 5.00
	Aspirin	3.5	3.00 ~ 4.00
	Corticosteroids	4	4.00 ~ 4.00
Lower urinary tract symptoms	Anticholinergics	4	3.50 ~ 4.25
	TCAs	4	4.00 ~ 4.25
	Chlorpromazine	4	4.00 ~ 4.25
Urinary retention	Anticholinergics	4	4.00 ~ 5.00
	TCAs	4	4.00 ~ 5.00
	Chlorpromazine	4	4.00 ~ 5.00
Benign prostatic hyperplasia	Anticholinergics	4	3.75 ~ 4.25
	TCAs	4	4.00 ~ 4.25
Urinary incontinence	TCAs	4	3.00 ~ 4.00
	Alpha-adrenoreceptor antagonists in women	4	4.00 ~ 4.00
Prostate adenoma	Anticholinergics	3.5	3.00 ~ 4.25
**Musculoskeletal system and connective tissue**			
Gout	Thiazide diuretics	4	3.75 ~ 4.00
Osteoarthritis	Corticosteroids	4	4.00 ~ 4.00
Osteoporosis	Corticosteroids	3.5	3.00 ~ 4.00
**Neoplasms**			
Breast cancer	Oestrogens	4	4.00 ~ 4.25
**Respiratory system**			
Chronic obstructive pulmonary disease	Benzodiazepines	3.5	3.00 ~ 4.25
	Beta blocking agents	3.5	3.00 ~ 4.00
	Corticosteroids	4	3.00 ~ 4.00
Asthma	Benzodiazepines	4	4.00 ~ 4.50
	Beta blocking agents	4	3.75 ~ 4.25
Respiratory failure	Benzodiazepines	4	3.00 ~ 4.25
**New PIMs suggested by the expert panel**			
Severe active liver diseases	Acetaminophen	4	4.00 ~ 4.00
Transplanted organ and tissue status	Fluoroquinolones	4	4.00 ~ 4.00

## Discussion

This study developed and validated the first explicit tool for assessing PIM use in older adults in Hong Kong. Many country-specific explicit tools have been developed by including all the PIMs defined by several previously published criteria and validated in the local context [29–31]. However, the current study selected the most common PIMs from the nine sets of reference criteria published in North America, Europe, and Asia, which enhanced the generalizability of the Hong Kong-specific PIM criteria for international comparisons. Furthermore, new PIM statements, which were not included in the previously published criteria, were also integrated into the Hong Kong-specific PIM list based on the local clinical experience.

Among the 164 statements classified as PIM use in the Hong Kong-specific PIM list, experts had agreed with approximately 70% of them after the first round of questioning. This result may indicate that strong evidence could prove the pharmacological inappropriateness of these statements in older adults or better therapeutic alternatives exist in the public sector in Hong Kong. By contrast, some of the statements were not designated as PIM use until the second round of rating, such as certain hypnotics and sedatives (zopiclone and zolpidem), which may be because the expert panel believed that these drugs could be used with low dose or in short term depending on individualized situations in the context of Hong Kong. The proton pump inhibitors (PPIs) were designated as non-PIM use after the first round of questioning in this study. It is probably because the experts think PPIs can be used with caution among elder patients as long as the therapy duration is not longer than 8 weeks. Another reason could be the experts believe the misuse of PPIs is not particularly problematic for older adults, while the development of the Hong Kong-specific PIM criteria is unique to the aging population.

After two rounds of the Delphi process, eight PIM candidates still remained questionable PIMs. Several reasons accounted for the questionable PIMs that the panel didn’t reach consensus on. First, some of the PIM candidates were regarded as inappropriate use in older adults in the reference criteria but no therapeutic alternatives existed in the HA drug formulary (e.g. fluoxetine as an antidepressant). Second, the expert panel decided that some of the questionable PIMs were acceptable for use in older adults as long as the dosage was not excessive (e.g. acetaminophen as an analgesic). Third, several disease-drug interaction candidates were rated as undecided or neutral because the expert panel felt it was necessary to balance the benefits and risks of the potentially inappropriate drugs depending on the individuals’ circumstances. According to the previous studies conducted in other countries [13, 14], questionable statements that experts did not reach consensus on should be generally considered as appropriate for use in elder patients. Hence, the questionable PIM candidates were excluded from the Hong Kong-specific PIM criteria in the current study.

Apart from the preliminary PIM list, the panel also proposed nine new PIM candidates, seven of which were eventually designated as PIM use. However, some of these newly suggested statements were only applicable to a subgroup of older adults, such as bisphosphonates to bedbound patients, and lacked of evidence from reference criteria. Therefore, more research findings are necessary to justify the inappropriateness of these seven PIM statements in the future updates.

Compared with the Beers criteria, the Hong Kong-specific PIM criteria are more user-friendly to guide the local prescribing practice. All the individual medications that appeared in the Hong Kong-specific criteria were listed with ATC classification codes, making it easier for health professionals to identify active substances. Therapeutic alternatives to PIMs independent of diagnoses are also offered to health professionals for reference. To be externally valid, explicit indicators for PIM use with adequate validity should be linked with adverse health outcomes. Some previous studies showed a positive association between the Beers criteria and multiple patient-related outcomes including hospitalization, mortality, falls and functional decline [32]. The relationship between the Hong Kong-specific PIM criteria and patient-related health outcomes needs to be validated externally in different settings in future studies.

The Hong Kong-specific PIM criteria can be used to assess prescribing patterns in Hong Kong and to educate patients and health professionals on appropriate drug use. The statements listed in the Hong Kong-specific PIM criteria should be generally avoided in older adults aged 65 years or above from a population level. However, the criteria cannot replace a clinician’s final decision-making from an individual level. If patients are not responsive to the therapeutic alternatives, the potentially inappropriate medications included in the criteria could also be prescribed for elder patients with close monitoring and dose adjustments under specific circumstances. The development of the Hong Kong-specific PIM criteria is intended to raise awareness of difficulties of pharmacotherapy for elder patients. Because all the medications included in the Hong Kong-specific PIM list are covered by the HA drug formulary, they can be integrated easily into the HA computer system and updated on a cycle.

The limitation of the Hong Kong-specific PIM criteria was that the preliminary PIM list was derived from previously published criteria with insufficient evidence on drug use from recent findings. The current study was intended to develop a disease-oriented PIM list and only two categories of PIM criteria were considered. Thus, expanding the Hong Kong-specific PIM criteria and including other categories of PIM criteria such as drug-drug interactions or duplications in future updates is necessary. Furthermore, the expert panel consisted mainly of physicians and pharmacists from public hospitals and the newly developed criteria were only applicable in the public sector. Thus, to facilitate the effects of the Hong Kong-specific PIM list, the selection of PIM candidates and the expert panel from the private sector should also be considered in the development of future versions of PIM lists.

## Conclusions

In conclusion, the Hong Kong-specific PIM list can be applied as a quality measure and an educational tool to improve the appropriateness of prescribing in the local context. It can provide physicians and pharmacists with practical advice to make better therapeutic decisions. Future studies should focus on validating the association between the Hong Kong-specific PIM assessment tool and adverse health outcomes in clinical and research settings.

## Supplementary Information

**Additional file 1:.** The full list of medications or medication classes with ATC codes included in PIMs considering specific medical conditions

**Additional file 2:.** Main concerns and possible therapeutic alternatives of the Hong Kong-specific potentially inappropriate medication list

## Data Availability

All data generated or analyzed during this study are included in this published article and its’ supplementary information files.

## Abbreviations

PIM: Potentially Inappropriate Medication Use
HA: Hospital Authority
ATC: Anatomical Therapeutic Chemical
IQR: Interquartile ranges
