# Supplementary material for: Development of an explicit tool assessing potentially inappropriate medication use in Hong Kong elder patients
Source: BMC Geriatr. 2021 Feb 2;21:98. doi: 10.1186/s12877-021-02024-0 (PMC7856727; doi:10.1186/s12877-021-02024-0)
Supplement: Supplementary file 1 — Additional file 1:. The full list of medications or medication classes with ATC codes included in PIMs considering specific medical conditions [file 12877_2021_2024_MOESM1_ESM.docx]

**Additional file 1. The full list of medications or medication classes with ATC codes included in PIMs considering specific medical conditions**

| **Medication class/Medication** | **ATC code** | **Medication class/Medication** | **ATC code** |
| --- | --- | --- | --- |
| **NSAIDs** | M01A | **Calcium channel blockers** | C08 |
| Indometacin | M01AB01 | **Dihydropyridine CCBs** |  |
| Sulindac | M01AB02 | Amlodipine | C08CA01 |
| Diclofenac | M01AB05 | Felodipine | C08CA02 |
| Ketorolac | M01AB15 | Nifedipine | C08CA05 |
| Piroxicam | M01AC01 | Nimodipine | C08CA06 |
| Meloxicam | M01AC06 | Lercanidipine | C08CA13 |
| Ibuprofen | M01AE01 | **Nondihydropyridine CCBs** |  |
| Naproxen | M01AE02 | Diltiazem | C08DB01 |
| Mefenamic acid | M01AG01 | Verapamil | C08DA01 |
| Celecoxib | M01AH01 | **Beta blocking agents** | C07A |
| Etoricoxib | M01AH05 | Pindolol | C07AA03 |
| **Aspirin** | B01AC06 | Propranolol | C07AA05 |
| **AChEIs** | N.A. | Sotalol | C07AA07 |
| Donepezil | N06DA02 | Nadolol | C07AA12 |
| Rivastigmine | N06DA03 | Metoprolol | C07AB02 |
| Galantamine | N06DA04 | Atenolol | C07AB03 |
| **Thioridazine** | N05AC02 | Acebutolol | C07AB04 |
| **Tricyclic Antidepressants** | N06AA | Bisoprolol | C07AB07 |
| Imipramine | N06AA02 | Esmolol | C07AB09 |
| Clomipramine | N06AA04 | Labetalol | C07AG01 |
| Trimipramine | N06AA06 | Carvedilol | C07AG02 |
| Amitriptyline | N06AA09 | **Antipsychotics** | N05A |
| Nortriptyline | N06AA10 | Chlorpromazine | N05AA01 |
| Doxepin | N06AA12 | Fluphenazine | N05AB02 |
| Dosulepin (dothiepin) | N06AA16 | Prochlorperazine | N05AB04 |
| **Alpha-adrenoreceptor antagonists** | C02, G04 | Trifluoperazine | N05AB06 |
| Prazosin | C02CA01 | Haloperidol | N05AD01 |
| Doxazosin | C02CA04 | Ziprasidone | N05AE04 |
| Alfuzosin | G04CA01 | Zuclopenthixol | N05AF05 |
| Tamsulosin | G04CA02 | Pimozide | N05AG02 |
| Terazosin | G04CA03 | Clozapine | N05AH02 |
| **Chlorpromazine** | N05AA01 | Olanzapine | N05AH03 |
| **Methyldopa** | C02AB | Quetiapine | N05AH04 |
| **Thiazolidinediones** | A10BG | Sulpiride | N05AL01 |
| Pioglitazone | A10BG03 | Amisulpride | N05AL05 |
| Rosiglitazone | A10BG02 | Lithium | N05AN01 |
|  |  | Risperidone | N05AX08 |
|  |  | Aripiprazole | N05AX12 |
|  |  | Paliperidone | N05AX13 |
| **Medication class/Medication** | **ATC code** | **Medication class/Medication** | **ATC code** |
| **Sedative hypnotics** |  | **Anticholinergics** |  |
| **Benzodiazepines** |  | Chlorpheniramine | R06AB04 |
| Clonazepam | N03AE01 | Cyproheptadine | R06AX02 |
| Diazepam | N05BA01 | Dexchlorpheniramine | R06AB02 |
| Chlordiazepoxide | N05BA02 | Dimenhydrinate | R06AA02 |
| Lorazepam | N05BA06 | Diphenhydramine | R06AA02 |
| Bromazepam | N05BA08 | Hydroxyzine | N05BB01 |
| Clobazam | N05BA09 | Promethazine | R06AD02 |
| Alprazolam | N05BA12 | Chlorpromazine | N05AA01 |
| Nitrazepam | N05CD02 | Clozapine | N05AH02 |
| Flunitrazepam | N05CD03 | Olanzapine | N05AH03 |
| Triazolam | N05CD05 | Trifluoperazine | N05AB06 |
| Midazolam | N05CD08 | Atropine | A03BA01 |
| **Other sedatvie hypnotics** |  | Propantheline | A03AB05 |
| Buspirone | N05BE01 | Amitriptyline | N06AA09 |
| Chloral hydrate | N05CC01 | Clomipramine | N06AA04 |
| Melatonin | N05CH01 | Doxepin | N06AA12 |
| Zolpidem | N05CF02 | Imipramine | N06AA02 |
| Zopiclone | N05CF01 | Nortriptyline | N06AA10 |
| **Corticosteroids** | H02 | Paroxetine | N06AB05 |
| Betamethasone | H02AB01 | Trimipramine | N06AA06 |
| Dexamethasone | H02AB02 | Flavoxate | G04BD02 |
| Fludrocortisone | H02AA02 | Oxybutynin | G04BD04 |
| Hydrocortisone | H02AB09 | Solifenacin | G04BD08 |
| Methylprednisolone | H02AB04 | Tolterodine | G04BD07 |
| Prednisolone | H02AB06 | Trospium | G04BD09 |
| Triamcinolone | H02AB08 | Benztropine | N04AC01 |
| **Metoclopramide** | A03FA01 | Orphenadrine | M03BC01 |
| **Bupropion** | N06AX12 | Disopyramide | C01BA03 |
| **Oestrogens** | G03C | Prochlorperazine | N05AB04 |
| Estradiol | G03CA03 | **Acetaminophen** | N02BE01 |
| Tibolone | G03CB01 | **Fluoroquinolones** | J01MA |
| **Thiazide diuretics** | C03AA | Ofloxacin | J01MA01 |
| Hydrochlorothiazide | C03AA03 | Ciprofloxacin | J01MA02 |
| **SSRIs** | N06AB | Levofloxacin | J01MA12 |
| Fluoxetine | N06AB03 |  |  |
| Citalopram | N06AB04 |  |  |
| Paroxetine | N06AB05 |  |  |
| Sertraline | N06AB06 |  |  |
| Fluvoxamine | N06AB08 |  |  |
| Escitalopram | N06AB10 |  |  |
