# Supplementary material for: Development of an explicit tool assessing potentially inappropriate medication use in Hong Kong elder patients
Source: BMC Geriatr. 2021 Feb 2;21:98. doi: 10.1186/s12877-021-02024-0 (PMC7856727; doi:10.1186/s12877-021-02024-0)
Supplement: Supplementary file 2 — Additional file 2:. Main concerns and possible therapeutic alternatives of the Hong Kong-specific potentially inappropriate medication list [file 12877_2021_2024_MOESM2_ESM.docx]

**Additional file 2. Main concerns and possible therapeutic alternatives of the Hong Kong-specific potentially inappropriate medication list**

Part 1. Hong Kong-specific PIM list: independent of diagnoses

| **Medication** | **Main concerns** | **Possible therapeutic alternatives** |
| --- | --- | --- |
| **Antispasmodics** | | |
| Propantheline | ❖Seldom use as antispasmodic | −Non-pharmacological treatment −Otilonium, mebevirine or holopon for abdominal pain |
| Atropine (excludes ophthalmic) | ❖Seldom use as antispasmodic | −Non-pharmacological treatment −Otilonium, mebevirine or holopon for abdominal pain |
| Metoclopramide | ❖Increase risk of extrapyramidal symptoms (EPS) ❖Especially avoid in the elderly with renal failure | −Stemetil, gravol, betahistine −Cinnarizine or dimenhydrinate PRN |
| **Long-acting sulfonylureas** | | |
| Glipizide | ❖Increase risk of hypoglycemia depending on diabetes mellitus (DM) status | −Metformin −Short acting oral hypoglycemic agents (OHA) |
| **Cardiac glycosides** | | |
| Digoxin | ❖Avoid as first-line for heart failure but acceptable to be used for later stage ❖Restricted to second-line therapy for atrial fibrillation (AF) or heart failure (HF) with dosage ≤ 0.125mg/day ❖Cautious in elders with renal failure | −Beta-blockers |
| **Antiarrhythmics** | | |
| Disopyramide | ❖Strong anticholinergic effect | −Beta-blockers (for rate control) −Class Ia antiarrhythmic drugs, e.g. procainamide, quinidine |
| Amiodarone | ❖Restricted to low dose | −Beta-blockers (for rate control) |
| **Antihypertensives** | | |
| Methyldopa | ❖On bottom of the list if antihypertensive drugs are needed | −CCB, ACEI / ARB |
| Clonidine | ❖Avoid as first-line antihypertensive | −CCB, ACEI / ARB |
| Alpha-blockers  −Prazosin  −Doxazosin  −Terazosin | ❖Avoid use as an antihypertensive | −CCB, ACEI / ARB |
| **Calcium channel blockers** | | |
| Nifedipine | ❖Avoid immediate release | −Other CCBs (e.g. amlodipine)  −Adalat® Retard (modified-release Nifedipine) |
| **Sex hormones** | | |
| Androgens  −Testosterone | ❖Use with low target and dose | −Non-pharmacological treatment |
| Estrogens  −Estradiol  −Tibolone | ❖Increase risk of stroke  ❖Topical use for menopausal symptoms with low dose | −Non-pharmacological treatment |
| **Urologicals** | | |
| Oxybutynin | ❖ Strong anticholinergic effect | −Mirabegron −Vesicare® (solifenacin) |
| Alpha-blockers  −Prazosin  −Doxazosin  −Terazosin | ❖Avoid use in women for urinary incontinence  ❖Close monitoring of postural hypotension if use in men for benign prostatic hyperplasia (BPH)  ❖Restricted to low dose use  ❖Restricted to sustained release dosage form or use as third-line antihypertensive therapy with compelling indication of BPH | −Other newer alpha-blockers (e.g. alfuzosin) |
| **Antibacterials** | | |
| Nitrofurantoin | ❖Avoid use in patients with renal impairment  ❖Restricted to short-term treatment of uncomplicated urinary tract infection (UTI) | −Other antibiotics (e.g. augmentin, septrin) |
| **Analgesics** | | |
| NSAIDs  −Indometacin  −Sulindac  −Diclofenac  −Ketorolac  −Piroxicam  −Meloxicam  −Ibuprofen  −Naproxen  −Mefenamic acid  −Celecoxib  −Etoricoxib | ❖Side effects on renal function and cardiac function  ❖Restricted to short-term use | −Non-pharmacological, paracetamol, prednisolone  −Tramadol for renal impairment patients |
| Opiods  −Methadone | ❖Increase risk of respiratory depression and cardiac arrhythmia  ❖Use for patients with history of opioid dependence or terminal illness/malignancy | −Paracetamol or tramadol for short-term use |
| **Muscle relaxants** | | |
| Orphenadrine  Baclofen  Tolperisone | ❖Seldom use as muscle relaxants  ❖Baclofen can be used for spasticity with caution | −Non-pharmacological treatment |
| **Antipsychotics, typical and atypical generation** | | |
| Chlorpromazine  Prochlorperazine  Trifluoperazine  Haloperidol  Ziprasidone  Pimozide  Clozapine  Olanzapine  Quetiapine  Risperidone  Aripiprazole  Paliperidone | ❖Increase risk of stroke and cognitive decline in patients with dementia  ❖Avoid use typical antipsychotics unless patients have harm behavior to self or others  ❖Avoid use atypical antipsychotics unless for schizophrenia or psychotic disorders | −Non-pharmacological treatment  −New generation of antipsychotics |
| **Hypnotics and sedatives** | | |
| Benzodiazepines  *Short- and intermediate- acting*  Lorazepam  Alprazolam  Triazolam  Midazolam  *Long-acting*  Clonazepam  Diazepam  Chlordiazepoxide  Bromazepam  Clobazam  Nitrazepam  Flunitrazepam | ❖Avoid use unless patients are not responsive to milder hypnotics  ❖Short-acting benzodiazepines (e.g. lorazepam) are more acceptable to be used comparing to long-acting benzodiazepines  ❖If use, restricted to short-term or PRN use | −Non-pharmacological treatment  −Antihistamines, zolpidem, zopiclone |
| Zopiclone  Zolpidem | ❖Avoid use unless not responsive to milder hypnotics | −Non-pharmacological treatment  −Melatonin |
| **Antidepressants** | | |
| Imipramine  Clomipramine  Trimipramine  Amitriptyline  Nortriptyline  Doxepin (>6mg) | ❖Strongly anticholinergic effect | −Newer SSRIs and SNRIs |
| **Antihistamines** | | |
| Chlorpheniramine  Cyproheptadine  Dexchlorpheniramine  Diphenhydramine  Promethazine  Hydroxyzine | ❖Anticholinergic effect  ❖Diphenhydramine and promethazine are acceptable to be used as cough syrup  ❖If use, restricted to short-term or PRN use | −Second generation of antihistamines (e.g. loratadine) or newer antihistamines |
| **Laxatives** | | |
| Sodium phosphate enema  Polyethylene glycol electrolyte powder | ❖May cause severe dehydration, electrolytes imbalance, hypotension or acute kidney diseases  ❖Avoid unless use for colonoscopy  ❖Avoid use for long term or in renal failure patients |  |
| **Drugs affecting bone structure and mineralization** | | |
| Bisphosphonates | ❖Bedbound elderly patients are of increased risk of gastrointestinal bleeding or peptic ulcer diseases | −Conservative treatment of osteoporosis |
| **Antitussive** | | |
| Codeine phosphate linctus | ❖Increase risk of CNS adverse effects, sedation and fall  ❖Acceptable to be used to suppress cough for short term | −Pholcodine or promethazine-containing antitussive |

**Abbreviations**

PRN = pro re nata, meaning "as needed"

CCB = Calcium channel blockers

ACEI = Angiotensin converting enzyme inhibitors

ARBs = Angiotensin II receptor blockers

NSAIDs = Nonsteroidal anti-inflammatory drugs

SSRIs = Selective serotonin reuptake inhibitors

SNRIs = Serotonin and norepinephrine reuptake inhibitors

BPSD = Behavioral and Psychological Symptoms of Dementia

CNS = Central nervous system

Part 2. Hong Kong-specific PIM list: considering specific medical conditions

| **Disease/Syndrome/Condition** | **Medications** | **Main concerns** |
| --- | --- | --- |
| **Blood and blood-forming organs** | | |
| Blood clotting disorders | NSAIDs | ❖Use with caution |
|  | Aspirin | ❖Restricted to low dose for antiplatelet use |
| **Cardiovascular** | | |
| Syncope | AChEIs | ❖Avoid use for syncope caused by bradycardia  ❖Restricted to medium-acting agent (e.g. pyridostigmine) for the treatment of myasthenia gravis  ❖Use with caution |
|  | Thioridazine | ❖Blackbox warning for QT prolongation |
|  | TCAs |  |
|  | Alpha-adrenoreceptor antagonists | ❖Increase risk of CNS side effects and hypotension |
|  | Chlorpromazine |  |
|  | Methyldopa | ❖Increase risk of CNS side effects and hypotension |
| Heart failure | Thiazolidinediones |  |
|  | NSAIDs | ❖May induce fluid retention |
|  | Nondihydropyridine CCBs | ❖May induce fluid retention ❖Can be used for rate control in patients with atrial fibrillation |
| Heart block | TCAs |  |
|  | Beta blocking agents | ❖May further disease heart rate |
| Cardiac arrhythmia | Antipsychotics | ❖Increase risk of QT prolongation |
| **Central nervous system** | | |
| Delirium | Sedative hypnotics* | ❖Restricted to hospitalized patients |
|  | Benzodiazepines | ❖Induce delirium episode ❖Restricted to hospitalized or critical care patients |
|  | Anticholinergics | ❖Avoid unless for short-term use (episodic for cold and flu) |
|  | Antipsychotics | ❖Restricted to hospitalized patients or second-line low dose therapy |
|  | Corticosteroids | ❖Avoid unless for anti-inflammatory use for short term ❖Restricted to critical care use |
| Parkinson disease | Antipsychotics | ❖Avoid, if use, prefer atypical antipsychotics |
|  | Metoclopramide | ❖Antagonize dopamine treatment or exaggerate Parkinson symptoms  ❖Cinnarizine or dimenhydrinate PRN use as alternatives |
| Dementia/ Cognitive impairment | Benzodiazepines | ❖High risk of delusion ❖Restricted to hospitalized patients |
|  | Anticholinergics | ❖Use as short term as possible |
|  | TCAs |  |
|  | Antipsychotics | ❖Restricted to second-line treatment of BPSD and psychogeriatric diseases ❖ Avoid, if use, prefer atypical antipsychotics |
| Falls | Sedative hypnotics | ❖Restricted to hospitalized/institutionalized patients |
|  | Thioridazine |  |
|  | Benzodiazepines | ❖High risk of delusion and fall ❖Restricted to hospitalized patients |
|  | TCAs |  |
|  | Antipsychotics |  |
|  | Opioids | ❖High risk of delusion and fall |
| Epilepsy/Seizures | Bupropion | ❖Dose-related seizure risk |
|  | Thioridazine |  |
|  | Antipsychotics | ❖Depending on seizure control. Acceptable to be used if needed. |
|  | Bupropion | ❖Dose-related seizure risk |
| Behavioral and psychological symptoms of dementia | Antipsychotics | ❖Avoid, if use, prefer atypical antipsychotics |
| Depression | Methyldopa | ❖Methyldopa aggravates depression |
| Lewy body disease | Antipsychotics | ❖Increase mortality. |
| Sleep apnea syndrome | Benzodiazepines | ❖Risk of respiratory depression |
| **Circulatory system** | | |
| Postural hypotension | TCAs |  |
|  | Dihydropyridine CCBs | ❖ Monitor side effect and acceptable to be used depending on cardiac disease status ❖Restricted to amlodipine or sustained-release dosage form |
|  | Alpha-adrenoreceptor antagonists | ❖Use other antihypertensive unless for benign prostatic hyperplasia ❖Restricted to second-line therapy and sustained-release dosage form |
|  | Chlorpromazine |  |
|  | Thioridazine | ❖Seldom use |
| Hypertension | NSAIDs | ❖Limit to short-term use ❖Restricted to short-term use in controlled hypertension |
| Raynaud disease | Beta blocking agents | ❖Depending on cardiac disease status |
| Venous thromboembolism | Oestrogens |  |
| **Endocrine, nutritional and metabolic system** | | |
| Hypokalaemia | Thiazide diuretics |  |
| Hyponatraemia | SSRIs |  |
|  | Thiazide diuretics |  |
| Hyperkalaemia | AChEIs | ❖With caution |
| Hypercalcaemia | Thiazide diuretics |  |
| Diabetes | Corticosteroids | ❖Limit to short-term use for anti-inflammatory ❖Restricted to critical care or short-term inhalational use  ❖If use, monitor DM status |
| **Eye and adnexa** | | |
| Glaucoma | Anticholinergics | ❖Contraindicated in angle-closure glaucoma ❖Use as short term as possible |
|  | TCAs | ❖Contraindicated in angle-closure glaucoma |
| **Gastrointestinal system** | | |
| Chronic constipation | Anticholinergics | ❖Use as short term as possible |
|  | TCAs | ❖High risk of constipation |
|  | Methyldopa |  |
|  | Opioids | ❖High risk of constipation |
|  | CCBs | ❖Depending on cardiac disease status |
| **Kidney and urinary tract** | | |
| Chronic kidney disease | NSAIDs | ❖Cause exacerbation of chronic kidney disease |
| Peptic ulcer disease | NSAIDs (Non-COX-2 selective agents) | ❖Can still be used if no alternatives available or diagnosed with peptic ulcer many years ago |
|  | Aspirin | ❖Use other antiplatelet agents first  ❖Use with gastric medications |
|  | Corticosteroids | ❖Avoid unless for replacement therapy  ❖Restricted to short-term use with proton-pump inhibitor prophylactic therapy and Group IV use in hospitalized patients |
| Lower urinary tract symptoms | Anticholinergics | ❖Cause urinary retention ❖Use as short term as possible |
|  | TCAs |  |
|  | Chlorpromazine |  |
| Urinary retention | Anticholinergics | ❖Cause urinary retention ❖Use as short term as possible |
|  | TCAs |  |
|  | Chlorpromazine |  |
| Benign prostatic hyperplasia | Anticholinergics | ❖Cause urinary retention |
|  | TCAs |  |
| Urinary incontinence | TCAs | ❖Can be used for treatment of overactive bladder/ stress incontinence |
|  | Alpha-adrenoreceptor antagonists in women | ❖Use other antihypertensive for substitution unless it is for ureteral calculi expulsion |
| Prostate adenoma | Anticholinergics | ❖Use as short term as possible |
| **Musculoskeletal system and connective tissue** | | |
| Gout | Thiazide diuretics | ❖Increase concentration of uric acid |
| Osteoarthritis | Corticosteroids | ❖Acceptable to be used for flare (short term) ❖Avoid oral. Acceptable to use intra-articular injections  ❖Restricted to hospitalized patients or critical care use |
| Osteoporosis | Corticosteroids | ❖Increase risk of osteoporosis.  ❖Avoid unless patients are not responsive to other anti-inflammatory drugs  ❖Restricted to hospitalized patients or critical care use  ❖Use as short term as possible |
| **Neoplasms** | | |
| Breast cancer | Oestrogens | ❖Carcinogenic potential  ❖Symptomatic treatment or topical oestrogens as therapeutic alternatives |
| **Respiratory system** | | |
| Chronic obstructive pulmonary disease (COPD) | Benzodiazepines | ❖Risk of respiratory failure ❖Restricted to hospitalized patients or critical care use ❖Avoid unless for the anxiety symptoms |
|  | Beta blocking agents | ❖Cause exacerbation of COPD |
|  | Corticosteroids | ❖Restricted to inhalation routes and Group IV in critical care use ❖Acceptable to be used for COPD exacerbations by injection or orally |
| Asthma | Benzodiazepines | ❖Risk of respiratory failure ❖Restricted to hospitalized patients or critical care uses |
|  | Beta blocking agents | ❖Cause exacerbation of asthma |
| Respiratory failure | Benzodiazepines | ❖Cause respiratory failure ❖Restricted to hospitalized patients or critical care use |
| **Unspecified** | | |
| Severe active liver diseases | Acetaminophen | ❖Reduced daily total dosage is recommended |
| Transplanted organ and tissue status | Fluoroquinolones | ❖Increase risk of tendinitis and tendon rupture in patients who have undergone kidney, heart, or lung transplants |

*****Refer to other sedative hypnotics except benzodiazepines.

**Abbreviations**

AChEIs = Acetylcholinesterase inhibitors

TCAs = Tricyclic antidepressants
